# Reference frames in spatial communication for navigation and sports: an empirical study in ultimate frisbee players

**DOI:** 10.1186/s41235-020-00254-1

**Published:** 2020-11-05

**Authors:** Steven M. Weisberg, Anjan Chatterjee

**Affiliations:** 1 Department of Neurology, Center for Cognitive Neuroscience, University of Pennsylvania, Philadelphia, PA 19104 USA; 2 Department of Psychology, University of Florida, Gainesville, FL 32611 USA

**Keywords:** Spatial cognition, Reference frames, Spatial language, Navigation, Individual differences, Spatial perception, Sports

## Abstract

**Background:**

Reference frames ground spatial communication by mapping ambiguous language (for example, navigation: “to the left”) to properties of the speaker (using a Relative reference frame: “to my left”) or the world (Absolute reference frame: “to the north”). People’s preferences for reference frame vary depending on factors like their culture, the specific task in which they are engaged, and differences among individuals. Although most people are proficient with both reference frames, it is unknown whether preference for reference frames is stable within people or varies based on the specific spatial domain. These alternatives are difficult to adjudicate because navigation is one of few spatial domains that can be naturally solved using multiple reference frames. That is, while spatial navigation directions can be specified using Absolute or Relative reference frames (“go north” vs “go left”), other spatial domains predominantly use Relative reference frames. Here, we used two domains to test the stability of reference frame preference: one based on navigating a four-way intersection; and the other based on the sport of ultimate frisbee. We recruited 58 ultimate frisbee players to complete an online experiment. We measured reaction time and accuracy while participants solved spatial problems in each domain using verbal prompts containing either Relative or Absolute reference frames. Details of the task in both domains were kept as similar as possible while remaining ecologically plausible so that reference frame preference could emerge.

**Results:**

We pre-registered a prediction that participants would be faster using their preferred reference frame type and that this advantage would correlate across domains; we did not find such a correlation. Instead, the data reveal that people use distinct reference frames in each domain.

**Conclusion:**

This experiment reveals that spatial reference frame types are not stable and may be differentially suited to specific domains. This finding has broad implications for communicating spatial information by offering an important consideration for how spatial reference frames are used in communication: task constraints may affect reference frame choice as much as individual factors or culture.

## Significance

Whether finding your gate at the airport, describing the location of your lost phone, or explaining where you live, accurately communicating spatial information is critical. There are two main ways (called *reference frames*) to describe spatial information. *Relative* descriptions use one’s facing direction (left, right); whereas *Absolute* descriptions use stable cues that do not change with facing direction (east, west). For small-scale spatial tasks, like the positions of objects, people from the USA and Europe use Relative reference frames (“take the cup to your left”). For large-scale spatial tasks, like navigation, people from the USA and Europe vary in whether they prefer body- or Absolute reference frames (“turn right” or “go east”). This presents a crucial question about how to design effective spatial descriptions: are spatial reference frame preferences for large-scale spaces stable within individuals? One difficulty in answering this question is that few tasks besides navigation require communication of large-scale spatial information. Team sports, which require coordination among people through effective spatial communication, offer an opportunity to bridge this gap. In a study on ultimate frisbee players, we find no individual preference for reference frame type across the two large-scale tasks, but robust differences in reference frame use across the two tasks. This result underscores the importance of task-specific constraints in effective spatial communication. Although we use sports as a target domain, we believe this result has implications for spatial communication in engineering, architecture, navigation, and the military.

## Background

Communicating spatial information, whether through human interaction or through verbal directions from a global positioning system, is vital but difficult. For one thing, spatial communication requires that the information provider and receiver adopt a common reference frame: a spatial representation in which objects are contained, ordered, oriented, located, or thought to move. Without a common reference frame, a specific direction like “to the left” is potentially ambiguous—to the left of what? To resolve this ambiguity, the information provider must establish a reference frame. Cognitive scientists (from philosophy, psychology, linguistics, and neuroscience (e.g., Danziger 2010; Majid et al. 2004) have theorized that reference frames can be specified in one of three primary ways: Relative (body-based and with respect to one’s facing direction: “take the street to *your* left”); Absolute (environment-based and with respect to a stable property of one’s surroundings: “travel west”); or Intrinsic (*object-based* and with respect to a property of an object: “travel in the direction the clocktower faces”). As these examples illustrate, communicating spatial directions for navigation can use any of these reference frame types. Here, we focus on Absolute and Relative reference frames, as these are more common in spatial navigation contexts. A key distinction between them is that Absolute reference frame specifications do not vary as a function of the orientation of the speaker (travel west), whereas Relative reference frame specifications do vary. (The street to one’s left is only to the west if one happens to face north. Turning to face south means traveling to the left would take you east).

Further complicating matters, individuals differ in their preference for specific reference frames (Ward et al. 1986), sometimes mixing reference frame types within the same description (Taylor and Tversky 1996). When communicating, a direction provider may prefer using an Absolute reference frame, but the direction receiver may prefer using a Relative reference frame. In addition, different spatial tasks or different environments may naturally elicit the use of one type of reference frame over the other (Li and Gleitman 2002). In the USA and Europe, instructing someone where they should reach for an object almost always elicits the use of a Relative reference frame (e.g., “take the cup on your left” rather than “take the cup to the southeast”).

Reference frame preferences vary in lab-based tasks. In a seminal set of studies, Brown and Levinson (1993) presented participants with an array of objects on a table (e.g., a cup on the participant’s left, a ball in the middle, and a pen on the participant’s right). The participant then rotated 180° and was instructed to recreate the array. Dutch speakers solved the task with a Relative reference frame, placing the objects in the same positions relative to their body (e.g., the cup on the left, then the ball, with the pen on the right). A group of Tenejepan-speaking individuals solved the same task using an Absolute reference frame, keeping the objects in the same positions relative to global north (i.e., because the participant rotated 180°, the cup would now be placed on the participant’s *right* and the pen on the *left*). Later research by Li and Gleitman (2002) revealed that variability in preference due to culture may be outweighed by variability in properties of the task itself. Li and Gleitman (2002) showed that changing the environment to include views of the outside world (rather than a bare lab room), or including a stable landmark on the table, increased the use of global-north-centered reference frames in people from the USA (who typically prefer a Relative reference frame in the task). Varying the parameters of the task revealed that reference frame preferences might not be stable—people can flexibly use one reference frame or another, depending on certain factors.

More recent work on individual differences shows within-person stability of reference frames in peripersonal space (defined as the space immediately surrounding an individual; Rizzolatti et al. 1997). Using schematic diagrams of people and non-oriented objects (like squares and circles), Beller et al. (2016) showed that German speakers generally prefer to *reflect* their frame of reference—that is, when viewing a person facing an object in a scene, German speakers will report that a ball between the person and the object is *in front of* the object, rather than *behind* the object. Regardless of the particular frame of reference adopted, the relevant point here is that, measured across trials and over time, people are consistent, maintaining their reference frame preference in a simple schematic task. One notable counter-example is the role of expertise as it correlates with frame of reference selection. In one experiment, German medical students selected a different reference frame than German law students, but only in a medical context. In a generic context, reference frame preference was stable across both groups (Hüther et al. 2016).

While much is known about reference frame preference in peripersonal space, little is known about the stability of reference frames preference for vista-scale space (Montello 1993). There is some evidence that Absolute reference frames created by the alignment of buildings (Marchette et al. 2011) or global north (Frankenstein et al. 2011; Weisberg et al. 2018a) allow individuals to rely on different information to orient themselves in space, and provides empirical support for the hypothesis that reference frame choices vary considerably. But this hypothesis has yet to be tested systematically.

Additionally, little is known about whether reference frame preferences are stable within individuals across domains and how they vary based on the properties of the task. One reason for this gap is that spatial navigation is one of few spatial tasks that people (in the USA and Europe) solve with both types of reference frames. One exception is the sport of ultimate frisbee, which, played at organized levels, uses both reference frame types to communicate defensive schemes.

Here, we consider individual variability between Absolute and Relative reference frames across two domains: spatial directions and sports. We hypothesize that individuals have proclivities toward one type of reference frame, which biases their comprehension of spatial directions in similar ways across domains. We also investigate whether there are domain-specific reference frame biases.

We explore whether reference frame preference in one domain, communicating spatial directions in a navigation context, correlates with reference frame preference in another, communicating a defensive strategy in a game called ultimate frisbee. In a set of pre-registered analyses, we predicted that preferring one type of reference frame in one domain would correlate with preferring the same type of reference frame in the other domain. Both domains use Relative or Absolute reference frames interchangeably. In navigation tasks, navigators often refer to Absolute reference frames that are stable with respect to the facing direction of the individual (e.g., north/south/east/west) or Relative reference frames that vary depending on the facing direction of the individual (e.g., right/left). Similarly, in ultimate frisbee, players can refer to a field-centered reference frame or a Relative reference frame when communicating about defensive strategy. In both domains, people claim to have strong preferences for using one reference frame over another. We tested whether reference frame preference in one domain correlates with preference for the same type of reference frame in the other.

First, we will briefly introduce modern ultimate frisbee strategy so the naïve reader can interpret the experimental design and analyses (for a more in-depth description see "Appendix 1: Ultimate Frisbee Primer"). Ultimate frisbee involves passing a frisbee to teammates with the aim of catching it in the endzone to score. Once a point is scored by either team, the two teams switch sides to play the next point. The disc is typically thrown from one side of the body (as a forehand, from a right-hander’s right) or the other side of the body (as a backhand, from a right-hander’s left). Modern defensive strategy in ultimate frisbee involves a “force” in which players whose team does not have the disc (defense) attempt to stop the team with the disc from throwing passes on one side of the field. This strategy effectively limits an offensive player to throwing the disc from one side of their body, which is equivalent to throwing the disc to one side of the field (nominally, home—the sideline where the defense’s equipment is, or away—the other sideline). These two descriptions are both used widely in ultimate frisbee communities to specify the same “force.” For example, a defensive strategist might say “force home, force forehand”—using Absolute *and* Relative reference frames interchangeably to specify the defensive alignment.

Force home/force away are always stable within a game and thus refer to directions that are invariant to the orientation of the defensive team with respect to the broader environment (e.g., which side of the field they are on). On the other hand, the terms force forehand/force backhand vary with respect to home and away each time the defensive team switches side (that is, when a defensive team faces one direction, if forcing forehand aligns with forcing home, then when the teams switch sides, forcing forehand aligns with forcing away). Thus, one set of force descriptions (home and away) are invariant to the facing directions of the teams situated on the playing field, while the other set of force descriptions (forehand and backhand) vary depending on the team’s facing directions.

In addition to testing our pre-registered predictions about stable individual reference frame preferences across both domains, we conducted a set of exploratory analyses to investigate whether reference frame preferences varied systematically as a function of the domain. The purpose of these analyses was to generate hypotheses about situations in which reference frame use might be stable within a task, and thus not suitable to investigating individual differences. These exploratory analyses can inform applications—like what type of verbal instructions or display to provide on a GPS, or how to communicate complex spatial maneuvers in other domains (e.g., sports and the military).

## Materials and methods

### Participants

We recruited ultimate frisbee players from the Philadelphia area using email messages to area ultimate clubs and leagues and by handing out flyers at ultimate frisbee events. Participants were invited to participate in an online study, which they completed at home, and would take 30–60 min. They could choose to be paid either a guaranteed $10 or have a one-in-five chance of winning $50 (each participant who chose the latter option was randomly selected to receive payment with 4:1 odds against). We offered two different modes of payment to encourage participants to return the required paperwork, which they might not be motivated to do if paid only $10. To keep their data private, we did not collect information on which payment method participants chose for later analysis.

Seventy-six participants responded. Of those, 58 (18 identifying as female) could be verified and had complete data (a participant was verified if all confirmation codes were entered correctly on all versions of the experiment). Seven participants self-reported as Asian, one as African American or black, and 48 as Caucasian or white. Six participants self-reported as Hispanic and one participant did not wish to report ethnicity or race. The mean age of the participants was 27.5 ± 8.1 years.

### Experimental materials

Materials, methods, and data are available on the Open Science Framework (https://osf.io/tv7g3/).

#### Reference frame task

**Fig. 1:**
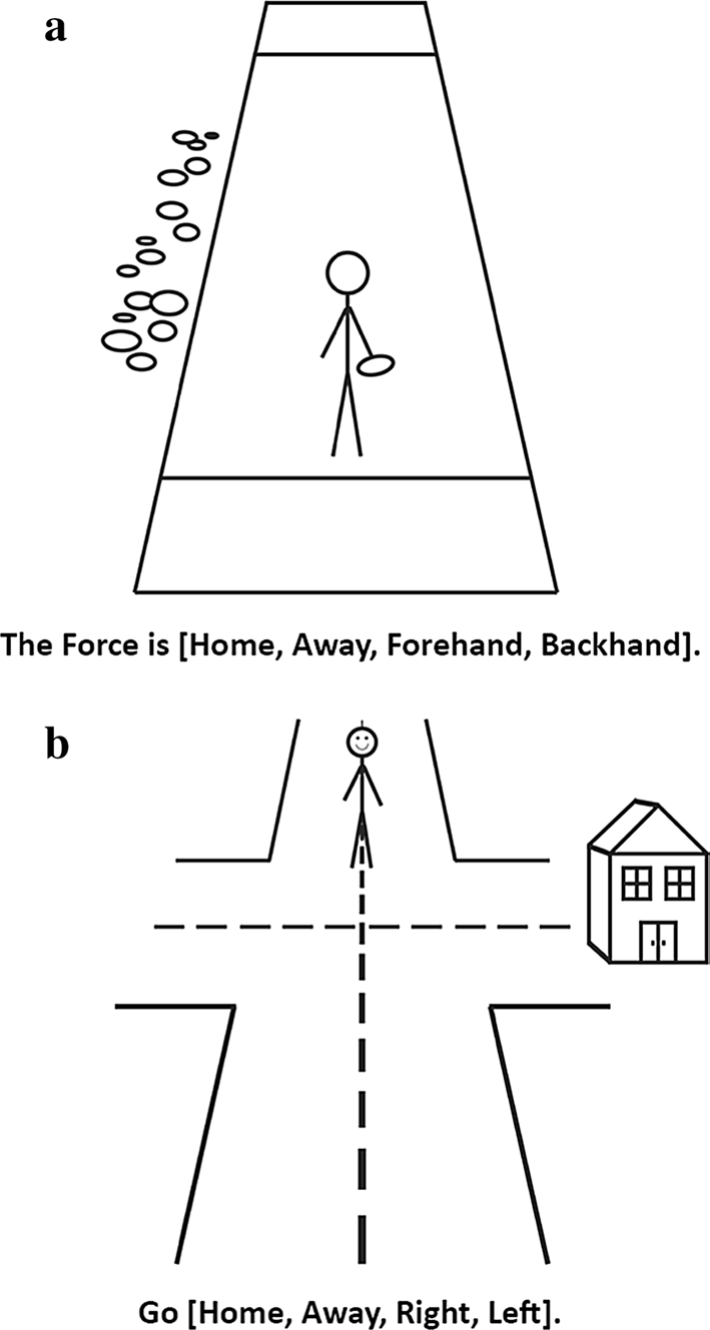
Ultimate frisbee and road intersection task stimuli. Stimuli used in the ultimate frisbee (**a**) and road intersection (**b**) task. The text that appears below each image are the four possible prompts that participants saw (with one word in brackets selected for each trial). In both tasks, participants were instructed to treat the location of home as either the sideline with visual clutter (ultimate frisbee) or the direction of the house (road intersection). Variables for the task were: prompt (the text that appeared); the location of “home” (left or right); and location of the stick figure [near side as in (**a**) facing away from the participant, or far side as in (**b**) facing toward the participant]

The reference frame task was designed in PsychoPy 1.85.2 (Peirce 2007, 2008) and administered online by exporting the experiment to HTML, which was hosted on a custom-built website. The reference frame task consisted of two parts, completed separately: a road intersection part and an ultimate frisbee part. Before each part, participants read through the instructions and saw seven sample trials with answers and completed eight practice trials with feedback. In the instructions, participants saw how to interpret the schematic figures (Fig. 1) and how to respond to various prompts. Participants could read through the instructions as many times as they needed.

In the road intersection part, participants viewed a four-way intersection with a stick figure in either the near ground (bottom of the screen, facing away from participants) or the far ground (top of the screen, facing towards participants). A house appeared on either the left or right of the intersection. For each trial, the image appeared followed by a 1-s delay, after which a prompt appeared at the bottom of the screen. Once the prompt appeared, participants were asked to respond by pressing the left or right arrow key on their keyboard as quickly as possible without sacrificing accuracy to indicate the direction the stick figure should go (to the left or to the right). Possible prompts were “Go Away,” “Go Home,” “Go Left,” and “Go Right.” Participants completed four randomized cycles through all possible combinations of trials (four prompts, two locations of the house [right or left], two positions for the stick figure [far or near]), resulting in 64 trials.

The ultimate frisbee part was identical to the road intersection part, except that the stick figure held a frisbee in its right hand and stood on an ultimate frisbee field. The stick figure stood in either the near endzone (bottom of the screen, facing away from participants) or the far endzone (top of the screen, facing towards participants). Visual clutter (small clusters of circles and ovals) appeared on either the left or right of the field, representing “home” (see “Ultimate Frisbee Primer”). For each trial, the image appeared followed by a 1-s delay, after which the prompt appeared at the bottom of the screen. Once the prompt appeared, participants responded by pressing the left or right arrow key on their keyboard as quickly as possible without sacrificing accuracy to indicate the direction of the force (i.e., which direction the defense should force the offense to throw the frisbee). Possible prompts were “The force is Away,” “The force is Home,” “The force is Backhand,” and “The force is Forehand.” Participants completed four randomized cycles through all possible combinations of trials (four prompts, two locations of home [right or left], two positions for the stick figure [far or near]), resulting in 64 trials.

To summarize the task, participants viewed an image and a prompt and had to respond by pressing either the left arrow key or the right arrow key to indicate in which direction the stick figure would go (or which direction the stick figure would be forced to go, in ultimate frisbee terms). We measured reaction time from the moment the prompt appeared until the participant responded. We also manipulated whether the stick figure appeared facing toward or away from the participant to ensure that Relative and Absolute prompts could not be predicted to be the same (e.g., both entailing a left response) across trials, but would vary depending on the stick figure’s facing direction. This manipulation was critical to ensure participants could not map left to home or right to away (or vice versa) automatically but would have to decide based on the stick figure’s facing direction.

Throughout this paper, we will use the following terms to describe trial types for the reference frame task: Far/Near refers to the position of the stick figure; Relative/Absolute refers to the type of prompt (specifying the reference frame as either relative to the facing direction of the stick figure or the absolute location anchored to home); and Left/Right refers to the position of “home” (on the left or right of the screen, not to the left or right of the stick figure).

#### Ultimate frisbee questionnaire

We asked a series of questions to assess participants’ ultimate frisbee playing and coaching experience, including years played, highest level played, preferred positions, and throwing ability. We also asked whether participants were comfortable calling the force as home/away or backhand/forehand, and which they preferred. We introduced this last set of questions after the first seven participants had already responded.

#### Santa Barbara Sense of Direction Scale (SBSOD; Hegarty et al. 2002)

This self-reported measure of navigation ability consists of 15 7-point Likert-scale items such as “I am very good at giving directions,” and “I very easily get lost in a new city.” The average score for each participant has been shown to correlate highly with performance on behavioral navigation tasks in real and virtual environments (Hegarty et al. 2002; Weisberg et al. 2014).

#### Debriefing and strategy questionnaire

We asked participants how they responded to each set of questions on the reference frame task and whether they experienced technical difficulties.

### Experimental procedure

The entire study took place on each participant’s personal computer (which was verified via automated device detection). Participants were directed to a website that contained a link to a Qualtrics survey. Participants began by waiving documentation of informed consent and then optionally providing their age, sex, gender, sexual orientation, ethnicity, and education (in years). Then, participants completed the SBSOD and the ultimate frisbee questionnaire. Next, participants were randomly assigned to complete the road intersection reference frame task or the ultimate frisbee reference frame task (random assignment across participants; due to chance and participant dropout, 26 participants completed the ultimate frisbee task first and 32 completed the ultimate frisbee task second. There were no differences on any tasks or subsets of tasks based on order of completion). The reference frame tasks were hosted on a separate website, containing the PsychoPy task. Once the first reference frame task was completed, they entered the confirmation code (unique to each participant and each task) on the Qualtrics survey, and then completed the second reference frame task. Finally, participants entered the confirmation code from the second reference frame task and completed the debriefing questionnaire. The confirmation code procedure was adopted to ensure that participants completed all elements of the study.

### Pre-registration

We pre-registered the main test of our hypothesis on Aspredicted.org (PDF available here: https://aspredicted.org/blind.php?x=uh86sc).

### Reference frame task data processing

As specified in the pre-registration, we first removed all participants who responded below chance or 2 standard deviations (SD) below the group mean for each task. After correcting accuracy (which was not specified in the pre-registration; see “Accuracy” in the “Results” section), no participants responded near chance and very few had accuracy 2 SD below the means (four for navigation, two for ultimate frisbee; the minimum performance was 82.8% for navigation and 81.3% for frisbee). The overall pattern of accuracy results suggests that participants understood and were engaged in the tasks. We analyzed data with and without these participants, but report data from all participants. As specified in the pre-registration, we removed all incorrect trials (278), as well as reaction times that were 2 SD slower than each participant’s average reaction time (343 trials). This procedure resulted in eliminating 600 trials, leaving 6824 trials (91.92%) for analysis.

These choices were made before seeing any data (as specified by the pre-registration). Because some choices in the pre-registration may have been misguided (e.g., eliminating inaccurate participants) and because other choices were arbitrary (2 SD reaction time threshold), we re-analyzed all main results including all participants and all trials (as well as other thresholds for reaction time trimming). We also analyzed results with non-right-handed ultimate players (*n* = 5) removed. The results of the study are robust to these choices. Additional statistics and figures can be generated using the Jupyter notebook here: https://mybinder.org/v2/gh/smweis/Ultimate/master.

### Statistics tools

Unless otherwise specified below, statistics were calculated using the scipy and numpy packages in Python (McKinney 2010; Oliphant 2006). Data were cleaned and processed with Pandas (McKinney 2010) and visualized using Matplotlib (Hunter 2007). Repeated measures analyses of variance (ANOVAs) were calculated using the ezANOVA package in R (version 4.4), using RStudio (RStudio Team 2016). Effect sizes are, for *t-*tests, Cohen’s *d*, and corrected for correlations for within-sample tests, and for ANOVAs, generalized eta squared (*η*^2^_g_) (Bakeman 2005).

## Results

### Pre-registered analyses

The main prediction from our hypothesis was that individual preferences for a Relative compared to an Absolute reference frame would correlate across the road intersection and ultimate frisbee tasks. Within each domain, we measured reference frame preference as the difference between average reaction time for body-centered (left/right or backhand/forehand) and Absolute prompts (Home/Away). Positive values on this measure can be interpreted as a tendency to respond more quickly for Absolute prompts, whereas negative values indicate quicker responses for Relative prompts. We predicted these differences would correlate within individuals across ultimate frisbee and road intersection tasks.

**Fig. 2:**
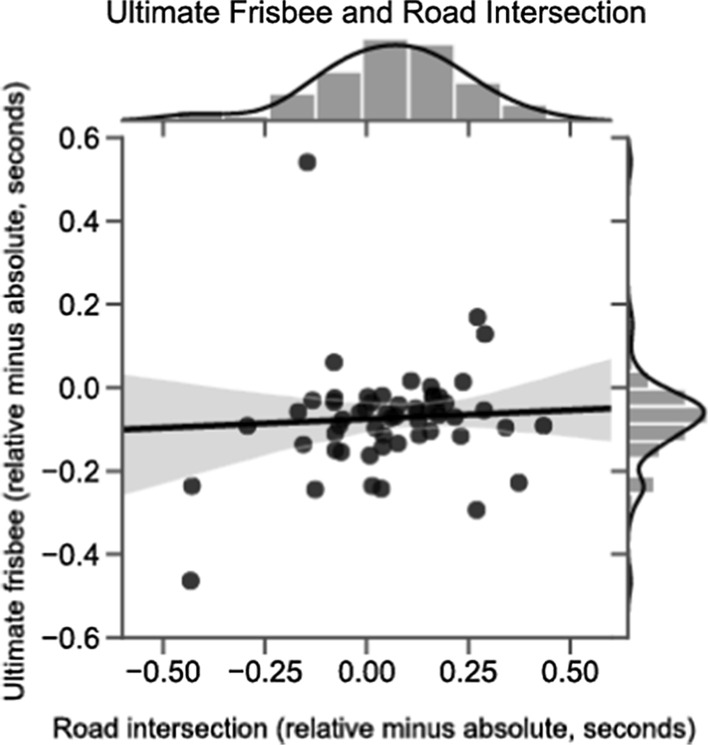
Correlation between reference frame preference across road intersection and ultimate frisbee tasks. Preference for a relative versus absolute reference frame was operationalized by the difference in reaction time between each trial type. Thus, negative values refer to faster reaction time on relative compared to absolute trials. As the scatter plot shows, there was no correlation between reference frame preference across the two task contexts

As shown in Fig. 2, there was no correlation, *r*(58) = 0.08, *p* = 0.56, between preference for Relative over Absolute reference frames in the ultimate frisbee and road intersection tasks. This result is below the threshold specified in our pre-registration (*r* = 0.22) for which we would run additional subjects. We fail to reject the null hypothesis. Converting this correlation coefficient to a *t* value (0.60) allows us to calculate the Bayes Factor (Rouder et al. 2009) as BF_01_ = 5.87 in favor of the null hypothesis. There is no systematic relation of preferences for reference frames between ultimate frisbee and road intersection parts.

**Fig. 3:**
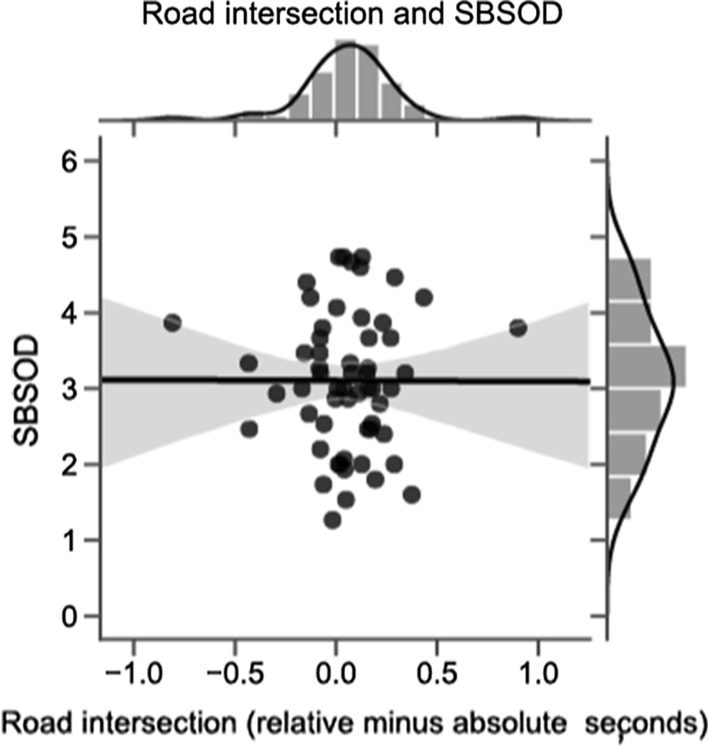
Correlation between navigation ability and reference frame preference on the road intersection task. Self-reported navigation ability (as measured by the Santa Barbara Sense of Direction [SBSOD] scale) showed no relationship to reference frame preference on the road intersection task. Better navigators did not respond more quickly to one reference frame type over another

**Fig. 4:**
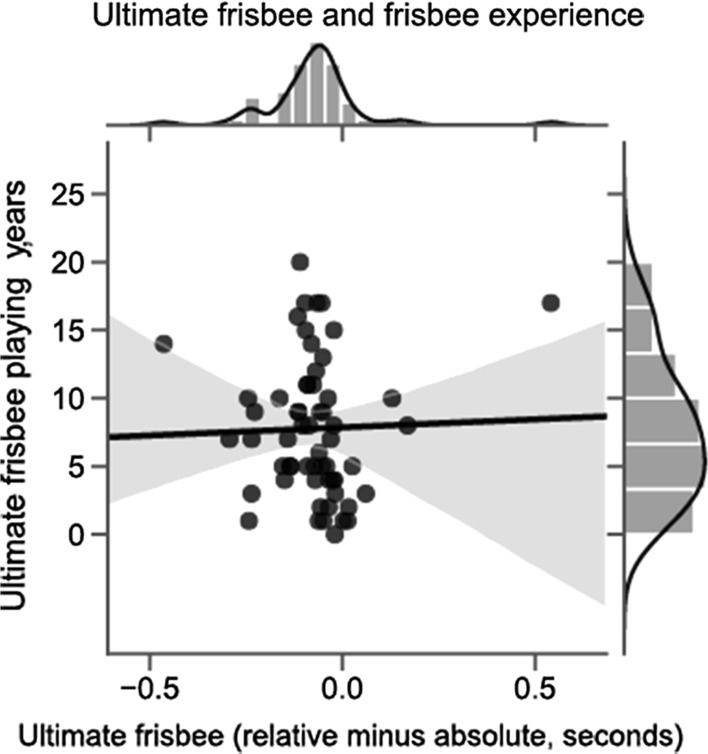
Correlation between ultimate frisbee experience ability and reference frame preference on the ultimate frisbee task. Years playing ultimate frisbee showed no relationship to reference frame preference on the ultimate frisbee task. This finding holds no matter which measure is used to determine ultimate frisbee ability

Our secondary analyses specified in the pre-registration were that navigation and ultimate frisbee ability, as measured by the self-reported questionnaires, would correlate with a preference for one reference frame or the other. As seen in Fig. 3, we correlated reference frame preference with SBSOD score for the road intersection task and found no correlation, *r*(58) = − 0.002, *p* = 0.98. The same pattern obtained for the ultimate frisbee task. As seen in Fig. 4, for ultimate frisbee, we used the number of years played for each participant as a proxy for experience and ability (although overall players were highly experienced, *M* = 7.78, SD = 5.03). This, too, resulted in no correlation, *r*(58) = 0.03, *p* = 0.82.

### Exploratory analyses

#### Accuracy

**Fig. 5:**
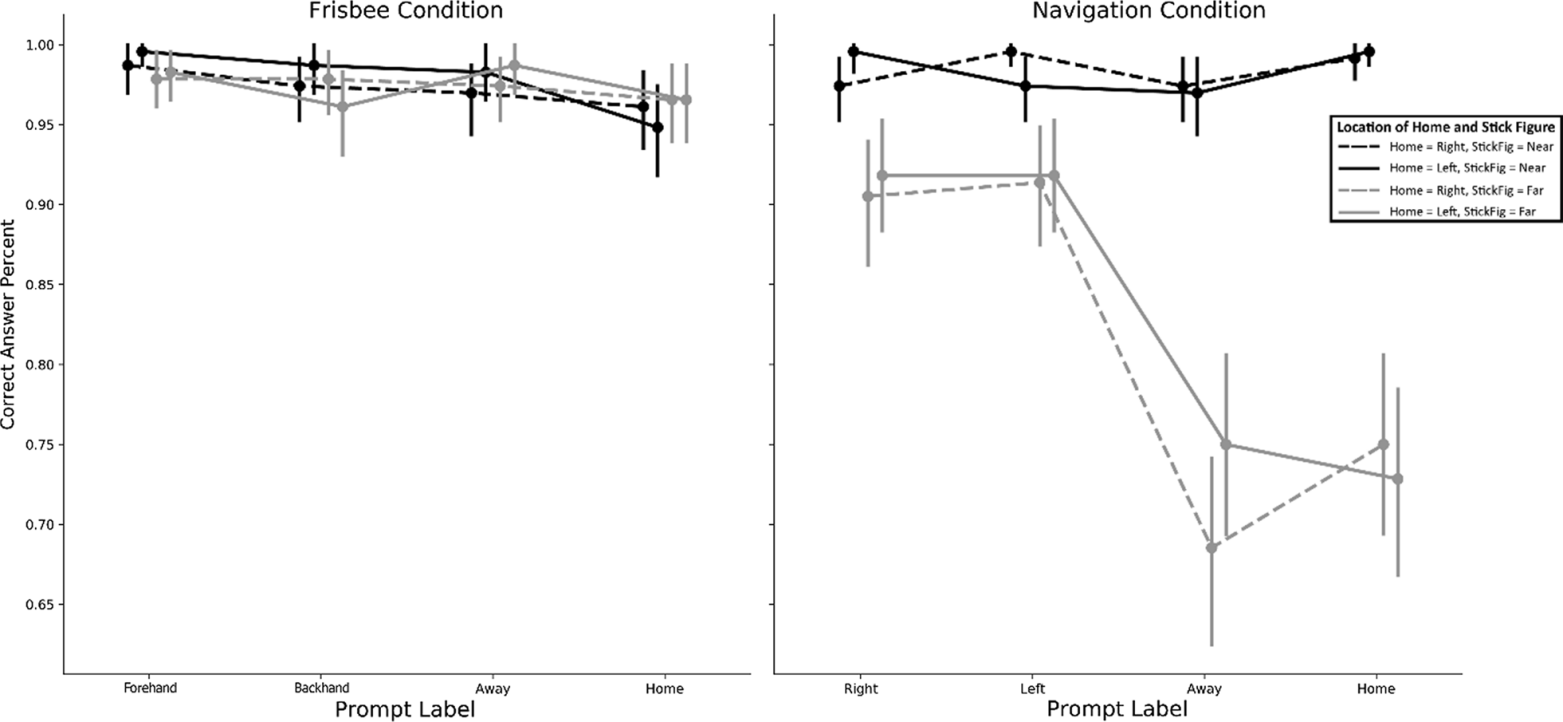
Accuracy differences across ultimate frisbee and navigation conditions. Across all participants, accuracy was high on the ultimate frisbee task. A subset of participants (flippers) responded distinctly lower for Absolute prompts on the road intersection task (navigation condition)

Accuracy was high on both tasks (ultimate frisbee: *M* = 97.5%, SD = 3.4%; road intersection: *M* = 90.2%, SD = 9.6%). To analyze accuracy, we used a three-factor repeated-measures ANOVA with condition (ultimate frisbee or road intersection), stimulus location (far or near), and prompt type (Absolute or Relative). This analysis (whose results are displayed in Fig. 5) revealed significant differences of condition, *F*(1,57) = 33.46, *p* < 0.001, *η*^2^_g_ = 0.06, stimulus location, *F*(1,57) = 45.87, *p* < 0.001, *η*^2^_g_ = 0.08, and prompt type, *F*(1,57) = 14.87, *p* < 0.001, *η*^2^_g_ = 0.03, characterized by all two-way and three-way interactions (all *p* values < 0.005). We explored these interactions, testing all possible pairwise contrasts and employing the Bonferroni correction for multiple comparisons (*α* = 0.05/28 = 0.002), and we found that the difference between conditions was driven mostly by poor performance on Far-Absolute road intersection trials (see Fig. 1b for this condition; *M* = 72.8%, SD = 37.5%) compared to all other trial types (all *p* values < 0.001 uncorrected). Additionally, relatively poor performance was seen on Far-Relative road intersection trials (*M* = 91.0%, SD = 9.0%) compared to all other trials (all *p* values < 0.001). The large standard deviation reveals large individual differences on Far-Absolute trials. In fact, 17 participants performed well below chance on these trials, with 14 participants answering fewer than 5 out of 16 correctly. Whereas two participants were borderline, answering nine trials correctly, 40 of the remaining 41 participants answered 15 or more trials correctly. No participants (including these 17) performed at or below chance on any other subset of trials for either task type. This bimodal distribution suggests a misunderstanding of the task for these specific trials.

This effect could not be due to a lack of exposure to those specific trial types. Between the practice trials and sample trials preceding each task, participants were exposed to all possible prompts, home positions, and stick figure positions, including the combination of Far-Absolute for the road intersection task.

We reasoned that this subset of participants misinterpreted the prompts for these trials. Instead of responding with the direction of Home or Away from their own point of view, these participants responded from the point of view of the stick figure (i.e., if the stick figure was on the far side of the screen, Home was on the left, and the prompt was “Go Home,” these participants would have responded “Right,” the direction the stick figure should go to get home, rather than “Left,” the direction the participant would see the stick figure go). We call this subset of participants Flippers because they flip their point of view to align with the stick figure.

##### Flippers and non-flippers

Overall, Flippers and Non-flippers did not differ on accuracy for trials that were not Far-Absolute on the road intersection task or the ultimate frisbee task (*p* > 0.25). Notably, Flippers were significantly slower on Absolute trials for the road intersection task, both Far (Flippers: *M* = 1.58, SD = 0.55; Non-flippers: *M* = 1.09, SD = 0.45, *t*(56) = 3.53, *p* < 0.001, *d* = 0.98) and Near (Flippers: *M* = 1.01, SD = 0.37; Non-flippers: *M* = 0.86, SD = 0.18, *t*(56) = 2.05, *p* = 0.04, *d* = 0.50). Reaction time did not differ on any of the ultimate frisbee task trials (all *p* values > 0.27) nor the relative road intersection trials (*p* values = 0.76). Flippers were also more likely to state a preference for an Absolute rather than a Relative reference frame when calling the force in ultimate frisbee, *χ*^2^(1) = 6.80, *p* = 0.009, Cramer’s *V* = 0.37. Despite differences, we elected to leave Flippers in for the reaction time analyses. Leaving them out does not alter the results.

#### Reaction time

For the reaction time analyses, we reverse-scored (i.e., marked trials that the participant responded to incorrectly as correct) Far-Absolute trials for participants who scored below chance (50%) on those trials. We trimmed reaction times that were 2 SD above the group mean of all reaction times, consistent with our pre-registered analyses, but left in incorrect trials after reverse-scoring the flipped trials for Flippers.

##### Comparing the ultimate frisbee and road intersection tasks

**Fig. 6:**
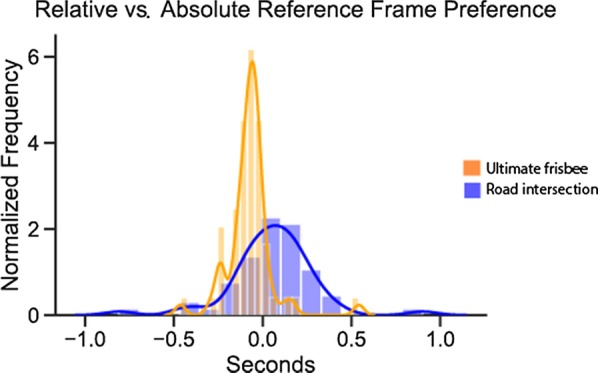
Different distributions of reference frame preference for the two task contexts. A histogram of relative minus absolute reference frame preference shows a greater preference on the ultimate frisbee task for a relative reference frame compared to an absolute reference frame preference for the road intersection task. The road intersection task also shows a wider spread, suggesting that individual variability may play a more critical role in that task context compared to ultimate frisbee (at least in this sample of ultimate frisbee players)

Like accuracy, reaction time analyses revealed systematic differences between the ultimate frisbee and road intersection tasks. In the ultimate frisbee task, participants were significantly faster for relative trials compared to absolute trials (*M* = − 0.10, SD = 0.08, *t*(57) = 9.71, *p* < 0.001). In the road intersection task, participants were significantly faster for absolute trials compared to relative trials (*M* = 0.06, SD = 0.18, *t*(57) = 2.59, *p* = 0.01). These preferences (displayed in Fig. 6) were also significantly different from each other, *t*(57) = 6.61, *p* < 0.001, *d* = 0.96. As can be seen in Fig. 6, the variance was significantly different between the ultimate frisbee and road intersection tasks (Levene’s test = 30.37, *p* < 0.001), with substantially greater variance in the road intersection task.

**Fig. 7:**
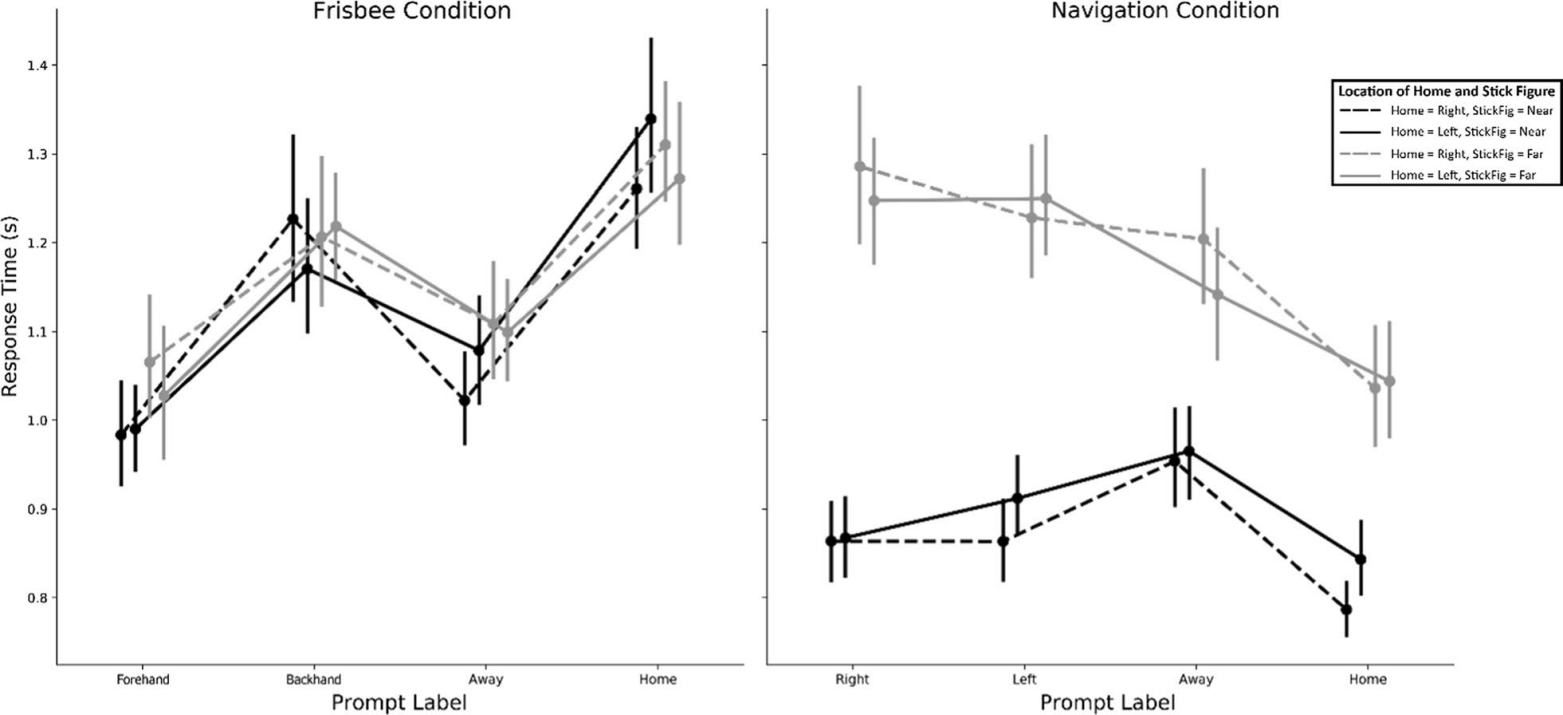
Reaction times by condition and trial type. Patterns of responses differed across the navigation and frisbee conditions. In the navigation condition, participants were sensitive to whether the stick figure was on the far side of the display, suggesting they were performing the task as if *they* were the stick figure. Conversely, they were not sensitive to the location of the stick figure in the frisbee condition. Instead, in the frisbee condition, the prompt type affected reaction time

**Fig. 8:**
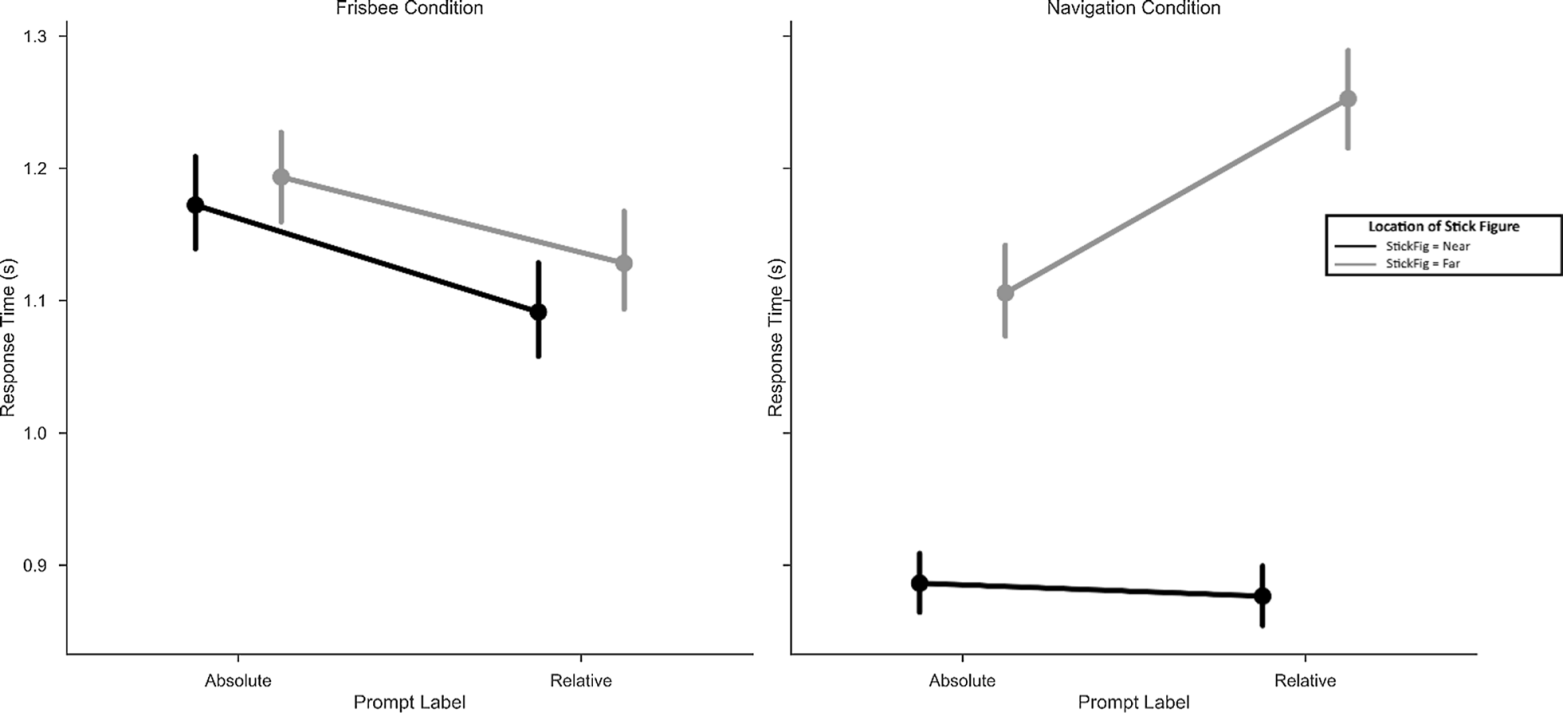
Reaction times by condition and trial type (absolute or relative). A simplified version of Fig. 7, which shows both the differences across frisbee and navigation conditions as a function of stick figure location, as well as the interaction between stick figure location prompt type in the navigation condition. For the navigate condition, participants had slower reaction times for relative trials compared to absolute trials when the stick figure was on the far side of the display, but had similar (and faster overall) reaction times when the stick figure was on the near side

Did performance vary as a function of trial type within both reference frame tasks? The answer to this question can be seen in Figs. 7 and 8. The following statistics summarize the main differences. We ran the same three-factor ANOVA as we did for accuracy—with condition (ultimate frisbee or road intersection), stimulus location (far or near), and prompt type (absolute or relative) as the three within-subject factors. This analysis resulted in significant main effects and interactions for all combinations of factors (all *p* values < 0.003). Road intersection trials (*M* = 1.03, SD = 0.20) were faster than ultimate frisbee trials (*M* = 1.13, SD = 0.25), *F*(1,57) = 18.11, *p* < 0.001, *η*^2^_g_ = 0.03. The largest effect was stimulus location. Near trials (*M* = 0.99, SD = 0.21) were faster than far trials (*M* = 1.17, SD = 0.23), *F*(1,57) = 231.23, *p* < 0.001, *η*^2^_g_ = 0.11. Finally, prompt type did not alter reaction times, with absolute (*M* = 1.09, SD = 0.22) and relative trials (*M* = 1.07, SD = 0.22) not detectably different, *F*(1,57) = 1.97, *p* = 0.17.

These main effects were qualified by two-way and three-way interactions (all interaction *p* values < 0.01). Specifically, the reaction time for road intersection trials was faster for near trials (*M* = 0.91, SD = 0.25) than for far trials (*M* = 1.30, SD = 0.46), *t*(57) = 10.10, *p* < 0.001 *d* = 0.81, whereas reaction for ultimate frisbee trials was more similar between near (*M* = 1.19, SD = 0.43) and far trials (*M* = 1.26, SD = 0.54), *t*(57) = 2.59, *p* = 0.01, *d* = 0.10. In addition, for the ultimate frisbee task, absolute trials (*M* = 1.28, SD = 0.47) were somewhat slower than relative trials (*M* = 1.17, SD = 0.50), *t*(57) = 5.23, *p* < 0.001, *d* = 0.17. For the road intersection task, absolute trials (*M* = 1.07, SD = 0.37) were more similar to relative trials (*M* = 1.15, SD = 0.36), *t*(57) = 2.17, *p* = 0.03, *d* = 0.16. To summarize, near trials on the road intersection task drove many of the two-way and three-way effects: participants were fastest when adopting a viewpoint consistent with their own. In contrast, the ultimate frisbee task was characterized by the reference frame imposed by the prompt, regardless of the stimulus location.

##### Stated frisbee preferences compared to reaction time frisbee preferences

Overall, nearly all ultimate players we asked indicated that they were extremely comfortable with both descriptions of the force (55 of 58 participants responded that they were at least moderately comfortable with home/away, and 56 of 58 participants responded that they were at least moderately comfortable with backhand/forehand). Nevertheless, out of the 51 participants we asked, 32 responded that they were more comfortable with home/away (absolute), whereas 19 responded that they were more comfortable with backhand/forehand (relative).

Despite stating different comfort levels with reference frames on the ultimate frisbee task, these groups did not differ on any measure of reaction time (or difference in reaction time) on the ultimate frisbee task. The two groups did differ on one aspect of the road intersection task. Participants who claimed to be more comfortable with absolute ultimate frisbee terms showed an advantage in responding to absolute trials on the road intersection compared to participants who stated a preference for relative ultimate frisbee terms, *t*(49) = 3.10, *p* = 0.003, *d* = 0.92. Given the exploratory nature of the finding and counter-intuitive result, we do not interpret this pattern.

## Discussion

The main goal of our study was to test the hypothesis that spatial communication preferences are stable within individuals across two domains: spatial navigation and ultimate frisbee. To our knowledge, this is the first study to compare reference frame preferences, using domains that can adopt different reference frames. In our pre-registered analysis, we did not find that preference for a specific reference frame in one domain correlated with preference in the other. Instead, despite surface similarities in the tasks, participants solved them differently. The reaction time data within each domain revealed that participants took the perspective of the stick figure during the navigation task but not in the ultimate frisbee task. Specifically, during the navigation task participants were sensitive to the location of the stick figure—slower to respond to Relative prompts when the stick figure faced away from them compared to when the stick figure faced towards them. There was no similar effect for Absolute prompts. Prompt type and stick figure location had no effect during the ultimate frisbee task, however, suggesting participants solved the task from a constant perspective.

Both domains elicited individual differences, though in different forms. The navigation task showed a broader spread of participant preference for Relative and Absolute reaction times than the ultimate frisbee task, which showed less variability overall and a more consistent participant advantage for Relative prompts. When asked about their preference explicitly for how to refer to the defensive scheme in ultimate frisbee, participants were split—17 preferred Absolute terms (“home/away”) whereas 32 preferred Relative terms (“backhand/forehand”).

One possibility for these observations is a conflation in design between reference frame preference with the ability to use a non-preferred reference frame. Although participants stated a reference frame preference when asked, almost all participants indicated that they were comfortable referring to the defensive scheme using either set of terms. (In fact, often when calling out the force, experienced players use both terms to mean the same thing: “Force forehand, force home.”) Nevertheless, despite this potential ambivalence, there was a notable overall advantage in reaction time using the Relative prompts for the ultimate frisbee task. Evidence that the two tasks are solved differently is further supported by the fact that a high proportion of participants accidentally “flipped” during the road intersection task—responding with the opposite response for absolute trials.

These findings reveal a task-specificity that is more consistent with Li and Gleitman’s (2002) data showing that people from the USA are sensitive to varying task parameters that alter their use of reference frame types than with Brown and Levinson’s (1993) interpretation of their data that people from the USA tend to prefer Relative reference frames broadly. These are also the first data to show evidence for flexible reference frame use across large-scale spatial tasks, rather than an emphasis solely on spatial navigation (e.g., Ward et al. 1986).

From a theoretical perspective, these data provide evidence that reference frame preference may not be a stable individual trait across tasks for large-scale spatial problems. Instead, considering whether a person is likely to take the perspective of another for a particular task predicts whether they will solve a task with one type of reference frame or another. Here, we are somewhat limited by the data. We observe that (at least some) participants adopted the perspective of the stick figure for the navigation task, even though, presumably, they could have solved the task without doing so. Even participants who did not flip left and right for the navigation task were slower in the navigation task for Relative prompts, suggesting the facing direction of the stick figure may have interfered in that case. This observation is similar to research on reference frames that show how variable people can be across tasks. Research on spatial reference frames reveals that, when communicating with others, speakers will flexibly adapt each others’ preferred reference frames (Johannsen and De Ruiter 2013a). Spatial reference frame selection is also dependent on the realism of the background scene (Johannsen and De Ruiter 2013b)—something we did not vary in the current experiment and, notably, distinguishes our results from real-world navigation and ultimate frisbee.

A potential ambiguity in interpreting these findings is the question of scale. We frame the navigation and ultimate frisbee domains as large-scale spatial problems but designed the task to take place on a computer screen. Although both domains operate in Montello’s (1993) vista-scale space, as they were tested, the task may actually tap small-scale resources. Little is known about the role of scale in spatial processing in general (e.g., how maps of space are converted into environmental-scale representations for use in navigation), but this is a limitation of the current design.

Variability in the adoption of perspectives from schematic depictions of environments has been reported before. In a study by Taylor and Tversky (1996), people studied maps of three different environments: a town; an amusement park; and a convention center. For the amusement park, people were split evenly in whether they provided descriptions using Absolute language or Relative language. But descriptions for the town and the convention center were more stable, with most people using Relative terms for the convention center but Absolute terms for the town.

In spatial perspective taking, work on American Sign Language (ASL) is of particular interest as location is typically coded iconically in ASL (Pyers et al. 2015) from the viewpoint of the signer, but easily decoded by experienced observers. That is, if a signer signs that a table is to their left, an observer understands this to mean to the *signer’s* left, not to their own left. In non-signers, people are sensitive to the viewpoint of others (Galati et al. 2013; Tversky and Hard 2009), incorporating available alternative viewpoints into their own descriptions of scenes. In the case of the ultimate frisbee task here, perhaps this viewpoint interference did not occur because participants represented the stick figure on the far side as on the opposing team.

The idea of taking another person’s perspective provides insight into another factor. In addition to a Relative or Absolute reference frame, Rock (1990) describes an object-centered reference frame for oriented objects. Consider the bicycle, which has a clear front and back. Stating “to the left of the bicycle” or “to the bicycle’s left” provides a reference frame independent of the other two. This type of reference frame may be used by participants who flipped the spatial responses from Relative (their own left) to object-centered (to the left of the other person). We did not consider this alternate reference frame in the design of the task, but it may play a critical role in supporting spatial communication.

Finally, this experiment has implications for understanding cross-modal representations. Research on the nature of representations of space in language, schemas (of which the diagrams here are one type), and images shows a common representation of spatial direction in the parietal lobe—a region of the brain thought to compute spatial directions from a Relative perspective (Weisberg et al. 2018b). This domain-specificity for neural computations has also been found across modalities, with distinct regions of the brain coding for actions and spatial prepositions (Amorapanth et al. 2012; Quandt et al. 2017). Generally, the way the brain seems to solve the cross-modality problem is by converting information into a common code for a particular domain. In the current experiment, we find that the translation between language and iconic representations (diagrams) differs depending on the particular task being solved, suggesting one might expect distinct neural involvement for the same type of task depending on whether recall is Absolute or Relative.

From an applied perspective, understanding how spatial reference frame use differs across tasks is a critical challenge for effective communication. Although spatial navigation is ubiquitous, it is not the only large-scale spatial task humans solve. In *Learning to Think Spatially* (National Research Council 2006), the authors describe the role of spatial thinking across a number of everyday domains, including architecture, air traffic control, and various sciences (including astronomy and the geosciences). To this list, one could add military operations, sports, urban and architectural design, and various types of engineering (perhaps civil and transportation engineering most prominently).

Knowing more about how to communicate spatial information effectively and quickly could allow for better coordination of one’s movements with others without a large cognitive burden, saving time, disorientation, and avoiding dangerous situations. Applying the knowledge from this experiment will require future research paradigms that capture the large-scale spatial characteristics of these tasks. Immersive virtual reality experiments would be an important next set of studies, which can more directly connect the sensorimotor aspects of the spatial task to the stimuli in a way that may change the results or make them more meaningful for spatial communication research.

By establishing a paradigm in which spatial reference frame use can be evaluated across domains, we hope more can be learned about the use of spatial reference frames in contexts where multiple people need to dynamically orient to their environment and to each other. The use of spatial reference frames in communicating through spatial language, maps, and visualizations remains poorly understood. One implication of the current work is that reference frame preference may not be stable within individuals across tasks and, in particular, certain reference frame types may be more readily understood by a large segment of the population than others. Disciplines with the goal of training and retaining spatial thinkers would be well-advised to consider how best to communicate spatial information given the specific tasks they face.

## Supplementary information

**Additional file 1: Figure S1.** Ultimate frisbee field and defensive strategy. An overhead view of an ultimate frisbee field with endzones at top and bottom, sidelines on the left and right (marked HOME and AWAY). The offensive team (*blue dots*) possesses the disc (*yellow dot*), while the defensive team (*red dots*) attempts to stop them from throwing and catching to each other. The defensive team does so by *forcing* the thrower to throw to one side of the field (the Open Lane, in *green*) and preventing the thrower from throwing to the other side (blocked by marker, in *red*). To communicate about the force, the defense could either specify which sideline to force the offense to throw to (force AWAY in this case) or they could call the force based on the throw a right-handed thrower would need to make (force FOREHAND in this case). Image courtesy of ultimatefrisbeeHQ.com.

## Data Availability

All data and materials (including data collection paradigms, instructions, experimental paradigm code, analysis code, and code for figures) are available on the Open Science Framework: https://osf.io/tv7g3/.

## Appendix 1: Ultimate frisbee primer

The purpose of this primer is to provide a brief overview of the sport of ultimate frisbee and to cover some of the main points of strategy, which are relevant to understanding the design of the study and interpreting our findings. Additional file 1: Figure S1 provides examples and diagrams that aid in interpreting this primer.

### General rules

Ultimate frisbee (named after the Frisbee—a circular disc that can be thrown, and flies, by spinning through the air) is a game played on a rectangular field by two teams of seven players each. The objective is to score points by catching the frisbee in the endzone. The frisbee can only be moved by passing it from player to player without dropping it and without it being caught by the other team. Players may not run with the frisbee. Defensive players may not take the frisbee away from an offensive player who is holding it. Once a point is scored by a player from one team catching the frisbee in the opposing team's endzone, the two teams switch sides and the scoring team throws off to the non-scoring team to begin the next point.

### Defensive strategy: the force

One prominent strategy for ultimate frisbee defense is to establish a *force*, so called because the strategy is to force the offense to throw the frisbee toward one sideline, while preventing them from throwing to the other. To do so, the defender who is guarding the player with the frisbee (the thrower) stands with their body in the way of the non-force side. All other defenders stand on the opposite side of the players they are guarding, to have the best chance of defending a throw that should come to that side of the field.

### Communicating the force

Establishing a force requires coordination among all defenders, and thus must be effectively communicated. By convention, two methods of communicating the *force* have arisen. One method, which specifies the force using the terms forehand and backhand, refers to the throw that a right-handed player should be forced to make. A forehand is thrown from the right side of a right-handed thrower’s body whereas a backhand is thrown from the left side of a right-handed thrower’s body. Another method, which specifies the force using the terms home and away, refers to the side of the field that an offensive player should throw toward.

Home refers to whichever sideline the players of both teams have left their belongings and away refers to the opposite sideline.

## Abbreviations

BF: Bayes’ factor
SBSOD: Santa Barbara sense of direction
