# Supplementary material for: Reference frames in spatial communication for navigation and sports: an empirical study in ultimate frisbee players
Source: Cogn Res Princ Implic. 2020 Nov 5;5:53. doi: 10.1186/s41235-020-00254-1 (PMC7644680; doi:10.1186/s41235-020-00254-1)
Supplement: Supplementary file 1 — Additional file 1: Figure S1. Ultimate frisbee field and defensive strategy. An overhead view of an ultimate frisbee field with endzones at top and bottom, sidelines on the left and right (marked HOME and AWAY). The offensive team (blue dots) possesses the disc (yellow dot), while the defensive team (red dots) attempts to stop them from throwing and catching to each other. The defensive team does so by forcing the thrower to throw to one side of the field (the Open Lane, in green) and preventing the thrower from throwing to the other side (blocked by marker, in red). To communicate about the force, the defense could either specify which sideline to force the offense to throw to (force AWAY in this case) or they could call the force based on the throw a right-handed thrower would need to make (force FOREHAND in this case). Image courtesy of ultimatefrisbeeHQ.com. [file 41235_2020_254_MOESM1_ESM.docx]

**
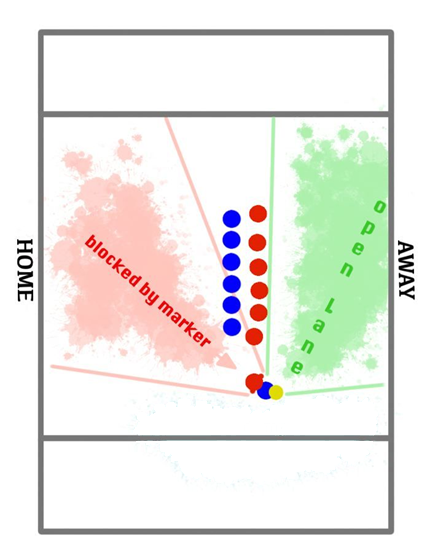
**

**Supplemental Figure 1.** Ultimate frisbee field and defensive strategy. An overhead view of an ultimate frisbee field with endzones at top and bottom, sidelines on the left and right (marked HOME and AWAY). The offensive team (blue dots) possesses the disc (yellow dot), while the defensive team (red dots), attempts to stop them from throwing and catching to each other. The defensive team does so by *forcing* the thrower to throw to one side of the field (the Open Lane, in green), and preventing the thrower from throwing to the other side (blocked by marker, in red). To communicate about the force, the defense could either specify which sideline to force the offense to throw to (force AWAY in this case), or they could call the force based on the throw a right-handed thrower would need to make (force FOREHAND in this case). Image courtesy of ultimatefrisbeeHQ.com.
